# Supplementary material for: Meta-Analysis and Gene Set Enrichment Relative to ER Status Reveal Elevated Activity of MYC and E2F in the “Basal” Breast Cancer Subgroup
Source: PLoS One. 2009 Mar 9;4(3):e4710. doi: 10.1371/journal.pone.0004710 (PMC2650420; doi:10.1371/journal.pone.0004710)
Supplement: Dataset S1 — GSEA results from first screen. The file contains a zipped archive of the HTML results of the second GSEA screen. Reports for enriched or depleted datasets may be accessed from the “index.html” link within the my_analysis.GseaPreranked.1219975950625.rpt.zip ZIP archive. Enrichment plots are available for each of the 50 most enriched and depleted gene sets. (13.32 MB ZIP) [file pone.0004710.s001.zip › my_analysis.GseaPreranked.1219975950625.rpt.zip!//HDACI_COLON_BUT12HRS_UP.html]

Details for gene set HDACI\_COLON\_BUT12HRS\_UP[GSEA]

|  || Dataset | zSco.ERneg.to.ERpos\_collapsed |
| Phenotype | NoPhenotypeAvailable |
| Upregulated in class | na\_neg |
| GeneSet | HDACI\_COLON\_BUT12HRS\_UP |
| Enrichment Score (ES) | -0.48530093 |
| Normalized Enrichment Score (NES) | -1.8405212 |
| Nominal p-value | 0.0 |
| FDR q-value | 0.027664758 |
| FWER p-Value | 0.642 |
Table: GSEA Results Summary

  

Fig 1: Enrichment plot: HDACI\_COLON\_BUT12HRS\_UP      
 Profile of the Running ES Score & Positions of GeneSet Members on the Rank Ordered List

  

| PROBE | GENE SYMBOL | GENE\_TITLE | RANK IN GENE LIST | RANK METRIC SCORE | RUNNING ES | CORE ENRICHMENT || 1 | SAT1 |  |  | 514 | 4.322 | 0.0179 | No |
| 2 | ANXA5 |  |  | 1144 | 3.315 | 0.0140 | No |
| 3 | MT1H |  |  | 2140 | 2.422 | -0.0292 | No |
| 4 | DNM3 |  |  | 2843 | 2.018 | -0.0556 | No |
| 5 | MT1G |  |  | 3092 | 1.889 | -0.0495 | No |
| 6 | F11R |  |  | 4089 | 1.477 | -0.1052 | No |
| 7 | MAP1LC3B |  |  | 4713 | 1.249 | -0.1357 | No |
| 8 | PPP3R1 |  |  | 4947 | 1.173 | -0.1379 | No |
| 9 | TUBB4 |  |  | 5352 | 1.025 | -0.1549 | No |
| 10 | BIRC4 |  |  | 6051 | 0.794 | -0.1970 | No |
| 11 | CAP1 |  |  | 6540 | 0.637 | -0.2254 | No |
| 12 | ROCK1 |  |  | 6704 | 0.585 | -0.2300 | No |
| 13 | UBE2H |  |  | 7143 | 0.438 | -0.2572 | No |
| 14 | INPP5E |  |  | 7393 | 0.347 | -0.2714 | No |
| 15 | FNBP4 |  |  | 8047 | 0.087 | -0.3194 | No |
| 16 | GSDML |  |  | 8057 | 0.085 | -0.3190 | No |
| 17 | GLRX |  |  | 8308 | -0.001 | -0.3378 | No |
| 18 | SERPINE1 |  |  | 8947 | -0.283 | -0.3821 | No |
| 19 | CLU |  |  | 9333 | -0.475 | -0.4049 | No |
| 20 | ATP6AP2 |  |  | 10103 | -0.909 | -0.4509 | No |
| 21 | C5 |  |  | 10561 | -1.215 | -0.4694 | Yes |
| 22 | WWOX |  |  | 10694 | -1.313 | -0.4621 | Yes |
| 23 | CDH1 |  |  | 10717 | -1.328 | -0.4464 | Yes |
| 24 | CTAGE5 |  |  | 11101 | -1.664 | -0.4534 | Yes |
| 25 | FUCA1 |  |  | 11484 | -2.016 | -0.4558 | Yes |
| 26 | GNAI2 |  |  | 11509 | -2.046 | -0.4308 | Yes |
| 27 | SERPINB1 |  |  | 11523 | -2.054 | -0.4048 | Yes |
| 28 | NEU1 |  |  | 11789 | -2.317 | -0.3944 | Yes |
| 29 | CREB3L1 |  |  | 11961 | -2.514 | -0.3744 | Yes |
| 30 | TSNAX |  |  | 12040 | -2.628 | -0.3458 | Yes |
| 31 | FOS |  |  | 12389 | -3.085 | -0.3316 | Yes |
| 32 | HIST1H1C |  |  | 12544 | -3.329 | -0.2996 | Yes |
| 33 | FLNB |  |  | 12764 | -3.781 | -0.2665 | Yes |
| 34 | CALCOCO1 |  |  | 12786 | -3.834 | -0.2179 | Yes |
| 35 | SPARCL1 |  |  | 12889 | -4.108 | -0.1718 | Yes |
| 36 | RHOB |  |  | 13091 | -4.795 | -0.1241 | Yes |
| 37 | TNCRNA |  |  | 13139 | -4.984 | -0.0623 | Yes |
| 38 | RHBDF1 |  |  | 13243 | -5.792 | 0.0058 | Yes |
Table: GSEA details [plain text format]

  

Fig 2: HDACI\_COLON\_BUT12HRS\_UP: Random ES distribution      
 Gene set null distribution of ES for **HDACI\_COLON\_BUT12HRS\_UP**
